# On the limits of Reactive-Spark-Plasma Sintering to prepare magnetically enhanced nanostructured ceramics: the case of the CoFe_2_O_4_-NiO system

**DOI:** 10.1038/s41598-019-50657-4

**Published:** 2019-10-01

**Authors:** Giulia Franceschin, Thomas Gaudisson, Nicolas Menguy, Raul Valenzuela, Frederic Mazaleyrat, Souad Ammar

**Affiliations:** 1 ITODYS, Université Paris Diderot, Sorbonne Paris Université, CNRS UMR-7086, 75013 Paris, France; 2 SATIE, ENS Cachan, Paris Saclay, CNRS UMR-8029, 94235 Cachan, France; 3 Sorbonne Université, CNRS UMR 7590, MNHN, IRD, Institut de Minéralogie, de Physique des Matériaux et de Cosmochimie, IMPMC, 75005 Paris, France; 4 IMM, Universidad Nacional Autónoma de Mexico, 04510 Mexico City, Mexico

**Keywords:** Magnetic properties and materials, Synthesis and processing

## Abstract

Magnetic materials are crucial for the efficiency of the conversion-storage-transport-reconversion energy chain, and the enhancement of their performance has an important impact on technological development. The present work explores the possibility of preparing hetero-nano-structured ceramics based on magnetic oxides, by coupling a ferrimagnetic phase (F) with an antiferromagnetic one (AF) on the nanometric scale. The field-assisted sintering technique or SPS (Spark-Plasma Sintering), adopted at this purpose, ensures the preservation of nano-sized crystals within the final solid structure. The aim is to establish how exchange bias may affect the resulting nano-consolidates and to investigate the potential of this process to increase the total magnetic anisotropy of the CoFe_2_O_4_ grains, and thus their coercive field, while keeping the saturation magnetization the same. The structure, microstructure and magnetic properties of the ceramics obtained were studied by several techniques. The results show that the sintering process, along with its typical reductive atmosphere, modifies the composition of the constituents. A new metallic phase appears as a consequence of the reciprocal diffusion of Co and Ni cations, leading to a change in the amount and structure of the AF phase. We propose a schematic representation of the atomic movements that hinder an exchange bias effect between the F and AF phases.

## Introduction

Over the last few decades, the increasing world requirements for more efficient and cheaper electronic devices have highlighted the need for advanced functional materials with energy-efficient fabrication and integration processes. Most of these consumer goods contain magnetic materials, which are essential components of energy, telecommunication, transportation, and medical applications, among others. The characteristics of the magnetic material employed play a fundamental role in the design for a specific application. For example, smaller devices, requiring lower electronic operating power, are successfully produced thanks to high-energy magnets, which allow their miniaturization.

Moreover, the integration of magnetically contrasted composite materials in such devices opens up the possibility of designing new systems with enhanced electromagnetic properties. These properties are, of course, derived from all the single constituents but also from their mutual coupling. For instance, coupling hard and soft ferromagnetic (F) phases or F and antiferromagnetic (AF) ones gives rise to exchange interactions that may impact the final properties in different ways.

The exchange interaction between F and AF nanomaterials, also known as exchange bias (EB) coupling, can enhance the thermal stability of small F structures^1,2^, overcoming their superparamagnetic limit^3^. It is thus used to increase the storage capacity of magnetic recording media, by making the bit grains as small as possible^4^. It is also exploited to stabilize the magnetization of the soft F reference layer in giant magnetoresistance (GMR)-based computer read heads^5–7^. Similarly, it is employed in commercial magnetic random access memory (MRAM) circuits^8,9^ and is being investigated for the design of rare-earth free permanent magnets^10^.

The origin of EB can be described in terms of an alignment of the AF spins at the F-AF interface, parallel to the F spins, occurring during a field-cooling event. The coupling between the AF and F spins at the interface exerts an additional torque on the F spins, which the external field has to overcome. Within this simple intuitive model, two opposite limiting cases can be predicted, depending on the strength of the AF magnetic anisotropy. If the AF anisotropy is large, EB should only induce a shift of the hysteresis loop, while if it is small, the only effect should be a coercivity enhancement^11^. Nevertheless, in general both effects are observed simultaneously, due to structural defects or grain size dispersion, which produce local variations of the AF anisotropy.

Currently, the most studied exchange-biased systems are thin films^12–19^ and granular systems^20–30^ in which the AF phase exhibits either large or small energy anisotropy, and the F and AF crystals share large common interfaces. Granular systems are mainly of three types: (i) F nanoparticles (NPs) embedded in an AF matrix, leading to the so-called embedded multicore morphology^3^; (ii) F NPs surrounded by an AF shell in a core-shell-like structure^20–24^, (or inversely AF NPs coated by a F shell)^25,26^ and (iii) nano-structured composite powders produced by ball-milling and made up of F and AF phases interpenetrated at the nanometer scale^27–30^.

Surprisingly, even if EB is expected to improve the hard magnetic properties of F-AF composite powders^31,32^, very little work has been done on consolidated solids to prepare permanent magnets. This deficiency is mainly due to the fact that sintering involves heating at high temperature, inducing grain growth and coarsening, significantly reducing the surface/volume atomic ratio and therefore the EB effect. Nevertheless, the recent advances in flash sintering techniques have propelled a renewed interest in consolidated hetero-nanostructures exhibiting EB. To the best of our knowledge, the only report of EB in a Spark-Plasma sintered material concerns a ceramic made from E-biased Fe_3_O_4_-CoO or composite NPs^33^.

Spark-Plasma Sintering (SPS)^34–37^ operates at relatively low temperatures and short sintering times, favoring ultrafine microstructures and high solid density^38,39^. It is also a powerful route for solid-state chemical reactions to form homogeneous^40,41^ or heterogeneous^42,43^ solids. Surprisingly it has been scarcely used to produce E-biased nanocomposite solids, apart from the previously cited work^33^.

In this context, we propose to tentatively elaborate E-biased, ultrafine-grained, magnetically contrasted consolidates by SPS co-sintering of F and AF nanoparticles. As F and AF NPs we chose two oxides, focusing on their ordering temperatures, T_C_ and T_N_, which should be as high as possible, and their magnetocrystalline anisotropy constants K_I_, which should be as different as possible. This choice will lead to a hysteresis broadening rather than to a hysteresis shift under field-cooling magnetic measurements, as a manifestation of EB at room temperature (r.t.). Spinel cobalt ferrite NPs CoFe_2_O_4_ (CFO) (T_C_ = 850 K and K_I_ = 1.2 10^3^ kJ.m^−3^ at r.t.) and rock salt nickel oxide NiO (NO) (T_N_ = 525 K and K_I_ = 10 kJ.m^−3^ at r.t.) were prepared with given sizes, typically 10 nm for the former and 5 and 30 nm for the latter. They were mixed at a 75:25 weight ratio and co-sintered at moderate temperature, namely 500 °C, for 5 minutes under a uniaxial pressure of 100 MPa.

In summary, the present work illustrates the preparation of sintered ceramics with nanoscale structuration and magnetic properties, and tries to understand the evolution of the magnetic properties after flash sintering. The ceramics are consolidated by the SPS technique, starting from magnetic nanopowders of F and AF phases, synthesized by the polyol method. The consolidation of an F phase in contact with an AF phase is expected to create interface coupling, called exchange-bias, which is related to an increment of the magnetic anisotropy of the resulting ceramic, as compared to the properties of the F phase in a nanoconsolidate sample. The high energy of the sintering process, along with the surface reactivity of the nanoparticles employed, is thought to provoke diffusion of the cationic species during sintering and to hinder the onset of EB.

## Results

### Structural and microstructural analysis of the ceramics

**Figure 1:**
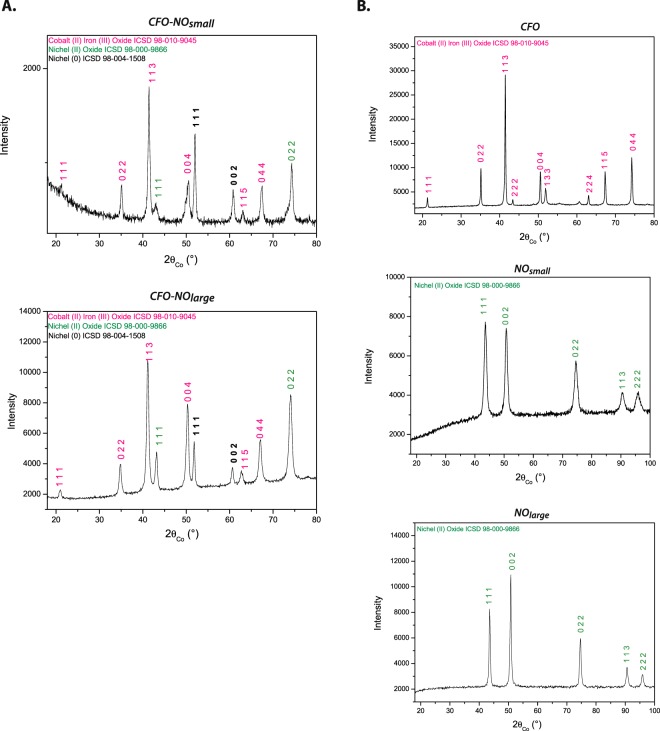
(**A**) XRD patterns of *CFO-NO* ceramics prepared from small and large NO particles. It must be pointed out that the large upwarp observed in the pattern of *CIO-NO*_*large*_ ceramic is an artefact. It is due to fluorescence effects. (**B**) XRD patterns of the single constituent phases. *CFO* and *NO* ceramics prepared from the corresponding nanoparticles.

X-ray diffraction was performed on all the ceramics produced, the bare CoFe_2_O_4_ (*CFO*) and NiO (*NO*) ones and their related *CFO-NO* composites. All the patterns exhibit quite narrow peaks (Fig. 1A,B), which are logically expected after the SPS process due to grain growth. We analyzed the data for the composite ceramics by Rietveld refinement (see Fig. SI-1 in the supporting information section), keeping in mind the characteristics of the reference ceramics.

Small and large NO particles gave ceramic materials characterized by a cell parameter equal to that of bulk NiO (Table 1) and an average crystal size no larger than 10 nm for the former and about 40 nm for the latter. This can be seen qualitatively from the diffractogram peak widths of this ceramic, which is noticeably broader. As for *NO*, *CFO* is characterized by a cell parameter equal to that of bulk CoFe_2_O_4_ (Table 1) and an average crystal size of about 40 nm. Whereas *NO* ceramics are pure, with no evidence of any metallic contamination, *CFO* exhibits a non-zero amount of fcc CoO metal precipitates in the form of large crystals. This feature is commonly observed in SPS sintered spinel ferrite ceramics^40,44–46^. Indeed, the sintering conditions might favor partial ferric cation reduction to the spinel lattice and cobalt de-mixing, leading to a chemical composition closer to Co^2+^_1-x_Fe^2+^_x_Fe^3+^_2_O_4_ than the initial Co^2+^_1_Fe^3+^_2_O_4_. This metallic contamination was also observed in the composite ceramic XRD patterns and was also assumed to originate from spinel de-mixing of transition metal elements.

**Table 1:** Main structural and microstructural properties of *CFO-NO* ceramics as inferred from Rietveld analysis, compared to those of pristine *CFO* and *NO* prepared separately as references.

	Spinel			Rock-salt			Fcc metal		
	a Å ± 0.005	<L_XRD_> nm ±2	Content wt.-% ±10	a Å ± 0.005	<L_XRD_> nm ±2	Content wt.-% ±10	a Å ± 0.005	<L_XRD_> nm ±2	Content wt.-% ±10
*NO*_*small*_	—	—	0	4.176	10	100	—	—	—
*NO*_*large*_	—	—	0	4.174	39	100	—	—	0
*CFO*	8.394	37	89	—	—	0	3.545	90	11
*CFO-NO*_*small*_	8.381	33	81	4.178	5	1	3.524	100^(*)^	18
*CFO-NO*_*large*_	8.389	20	68	4.200	38	22	3.540	100^(*)^	10

*These values were fixed during MAUD refinements, due to the very large crystal size (more than one hundred nm) of the related phase.

Starting from large NO NPs, the resulting ceramic preserves approximately the same AF phase weight ratio as that of the mixture introduced into the die before sintering (Table 1).

The refined cell parameters of its spinel and rock-salt phases were found to be very close to those of bulk CoFe_2_O_4_ and NiO, respectively, while that of its metallic contamination was found to be closer to that of bulk Co than bulk Ni. These results corroborate our expectations. The metal contribution arises mainly by cobalt de-mixing from the spinel phase, as in pristine *CFO*. On the other hand, the ceramic obtained from smaller NO NPs consists mainly of the spinel and metallic phases. Only a trace of a rock-salt phase was detected. The lattice parameter of the spinel phase is smaller than that detected for *CFO-NO*_large_ ceramic and is between the typical values for bulk NiFe_2_O_4_ and CoFe_2_O_4_. Moreover, the lattice parameter of the rock-salt phase is larger than that of bulk NiO, and that of the metallic phase has a value comparable to that of bulk Ni°. It appears that the size of the NO NPs, used to produce the ceramic material, has a significant influence on the stability of the oxide phase during the SPS process.

**Figure 2:**
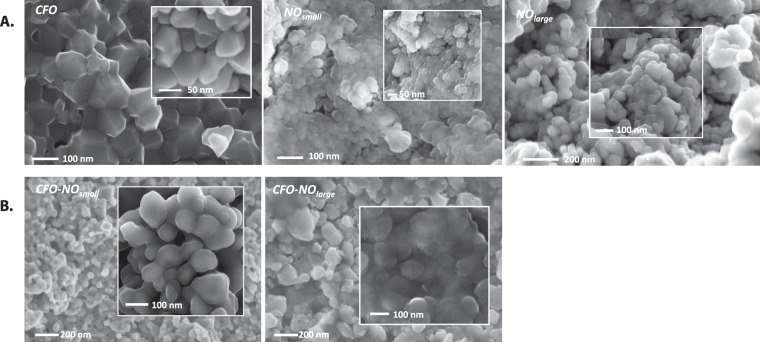
(**A**) SEM top view of *CFO*, *NO*_*small*_ and *NO*_*large*_ reference ceramics. (**B**) SEM top view of *CFO-NO*_*small*_ and *CFO-NO*_*large*_ composite ceramics.

**Figure 3:**
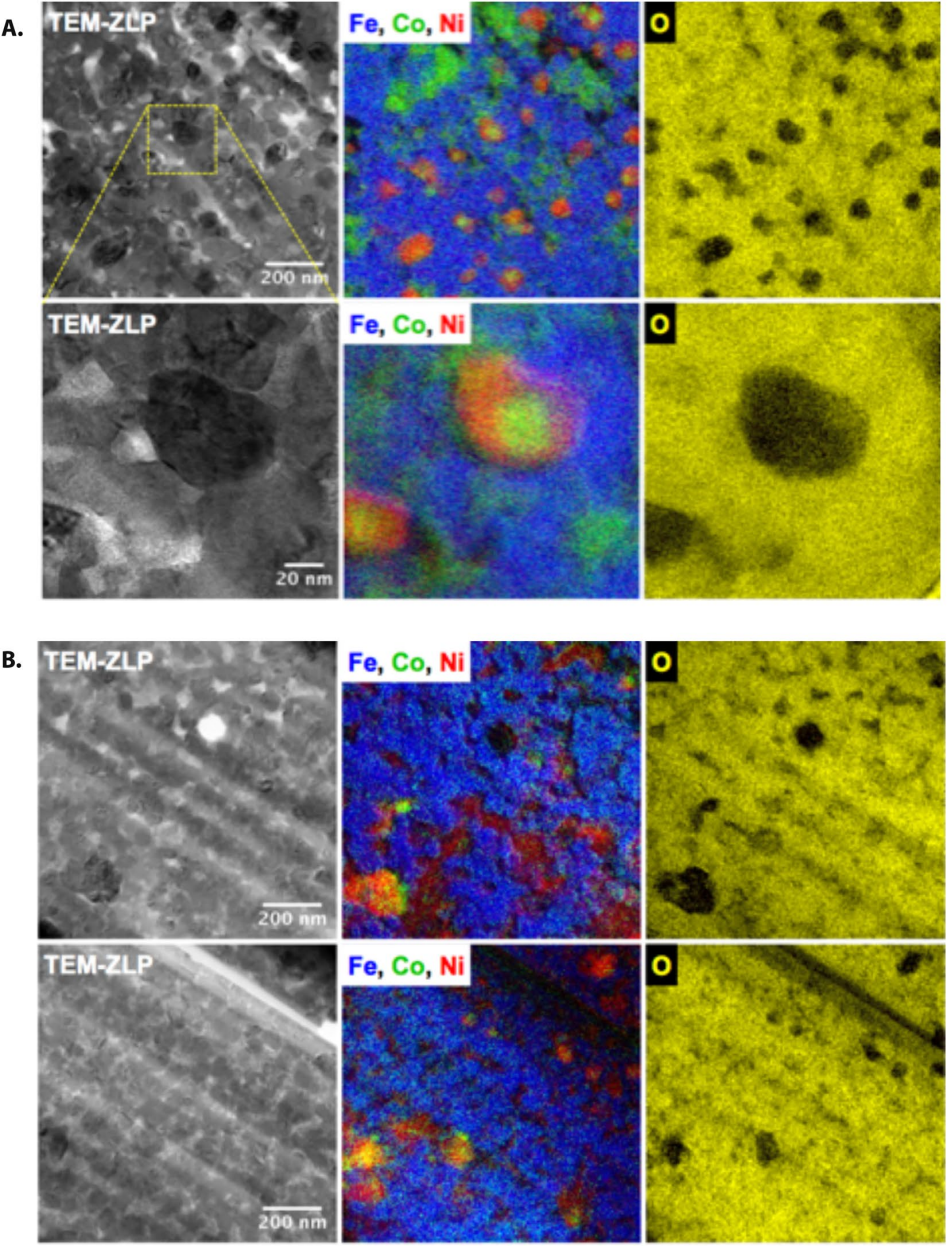
(**A**) *CFO-NO*_*small*_ elemental mapping performed on the same areas at different magnifications. On the left, TEM-ZLP bright field images show an overview of the sample; in the middle, Fe (blue), Co (green) and Ni (red) EFTEM mapping are overlapped; on the right, oxygen elemental mapping (yellow) highlights the O-poor zones, which correspond to metallic grains. (**B**) *CFO-NO*_*large*_ elemental mapping performed on two different areas (top) and (bottom). On the left, TEM-ZLP images show an overview of the sample; in the middle, Fe (blue), Co (green) and Ni (red) mapping are overlapped; on the right, oxygen elemental mapping (yellow) highlights the O-poor zones, which correspond to metallic grains.

Our investigations were completed by Scanning Electron Microscopy (SEM) (Fig. 2A,B) and Transmission Electron Microscopy (TEM) (Fig. 3A,B) observations. The SEM analysis showed that SPS processing allowed the production of highly dense ceramics with ultrafine grains in both small and large *NO*-derived composite ceramics. The average size of each ceramic was determined on the basis of a statistical analysis of a minimum of one hundred grains on selected SEM micrographs (see Fig. SI-2 in the supporting information section). It is found to be globally smaller than 100 nm.

TEM and Energy-Filtered TEM (EFTEM) analysis of the composite ceramics exhibit certain similitudes in their microstructure but also a lot of differences. In both cases, a metallic phase is formed by sintering (Fig. 3A,B). As a common feature, the chemical mapping of the two composite presents several O-depleted zones corresponding to metallic grains. Also, all the size of the metallic grains is in the submicrometer range, apparently larger than that of the grains in the O-rich zones. But whereas, the O-depleted zones in *CFO-NO*_large_ correspond to (Ni,Co) solid-solution grains, those in *CFO-NO*_small_ present a core-shell morphology with Co° as the core and Ni° as the shell.

In addition, *CFO-NO*_large_ ceramic shows a microstructure developed like a matrix of spinel ferrite including the metallic grains and some rock-salt-based areas of less than 100 nm in size. The chemical mapping indicates that both Co and Fe are detected in the spinel phase while both Co and Ni are detected in the rock-salt phase. These observations mean that the former cubic phase is composed of CoFe_2_O_4_ while the latter is mainly made by Ni_1-x_Co_x_O solid solution. Apart from the amount of reduced Ni and Co in the metallic grains, *CFO-NO*_large_ appears to have almost the same composition than that of its starting nanopowders.

In contrast, *CFO-NO*_small_ has a spinel matrix containing Ni, Co and Fe that together form Co_1-x-y_Ni_y_Fe_2_O_4_. Metallic grains are dispersed in this matrix. Their core-shell morphology is quite homogeneous, with an average diameter of about 100 nm. No evidence of the rock-salt phase is reported in the chemical mapping of this sample (Fig. 3A).

In Fig. 3A,B, the TEM-ZLP images (see the experimental section) evidence a more homogenous microstructure for *CFO-NO*_large_. Its grains are constant in shape and uniform in size. The greater size of the NO NPs introduced before sintering probably plays a role on keeping NiO more stable during the whole high-energy consolidation process.

### Magnetic characterization

Both *CFO-NO*_small_ and *CFO-NO*_large_ samples were studied from the magnetic point of view in an attempt to detect an EB effect between their F and AF phases. The results show important differences between the samples, which can be ascribed to the stability of their *NO* component.

**Figure 4:**
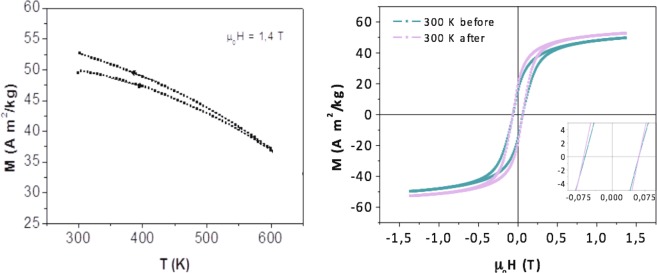
M(T) curves (left) recorded during a heating to 600 K and a cooling to 300 K cycle of *CFO-NO*_small_. R.t. M(H) loops obtained on the sample before and after heating to 600 K under 1.4 T magnetic field.

In Fig. 4, there is a slight irreversible thermal variation of the magnetization of *CFO-NO*_small_ between the in-field heating and in-field cooling plots. The heating and cooling curves are not exactly overlaid, the former exhibiting a higher magnetization than the latter. This feature was much more pronounced in the ceramic produced by small NO particles than that obtained from the larger ones. It is attributed to a partial reduction of the antiferromagnetic NiO phase in favor of the formation of a non-zero amount of ferromagnetic Ni° in the magnetometer. This feature was also observed in the hysteresis loops plotted in Fig. 4 (right). The loop recorded at r.t., before heating, has a lower saturation magnetization M_s_ than that recorded after field cooling from 600 K. An inflection point at about 630 K is seen in these curves. This temperature is coherent with the Curie temperature T_C_ of metallic Ni. The inflection point is clearly present in the cooling curve of small NO particles, while it is barely noticeable in that of large ones. We should note that in the case of *NO* ceramics, the Ni content does not exceed 2 wt.-%, so it can be neglected in the interpretation of our further magnetic investigations.

**Figure 5:**
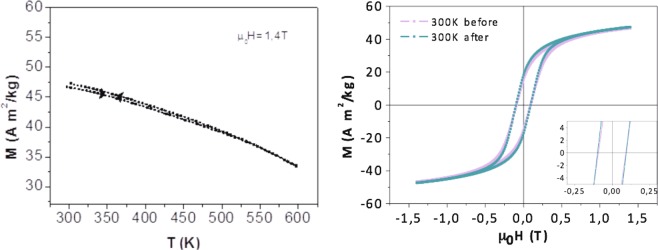
M(T) curves (left) recorded during a heating to 600 K and cooling to 300 K cycle of *CFO-NO*_large_ ceramic. R.t. M(H) loops obtained on the sample before and after heating to 600 K under a 1.4 T magnetic field.

For *CFO-NO*_large_, the thermal magnetization is much more reversible during the heating and cooling processes. The M(T) curves obtained are almost overlaid (Fig. 5 left), meaning that the suspected Ni° contamination is very weak, and certainly weaker than that formed in *CFO-NO*_small_. As a consequence, the hysteresis loops recorded at r.t. before and after heating to 600 K under a 1.4 T external field, are superimposed (Fig. 5 right), confirming that the composition of the sample does not evolve during the magnetic measurement.

The comparison of these loops gives additional information. For both the composite ceramics, there is neither broadening nor displacement along the field axis. In other words, there is no exchange-bias feature. This is true for both ceramics, suggesting that the relevant variation of chemical composition occurring during SPS sintering completely alters their magnetic behavior. The AF phase is in fact almost absent in the case of *CFO-NO*_small_, and is structurally modified in *CFO-NO*_large_, with a chemical formula approximating a cobalt-rich rock-salt Ni_1-x_Co_x_O solid solution.

The results obtained will be correlated in the following part in an attempt to better understand the behavior of the samples prepared. The more relevant values inferred from the magnetic measurements are shown in Table 2 for both the composite ceramics and pristine *CFO*.

**Table 2:** Values of the main magnetic characteristics of the *CFO-NO* ceramics as inferred from magnetic measurements and compared to those of pristine *CFO*.

	300 K, 1.4 T *Before* heating in field			300 K, 1.4 T *After* heating in field		
	µ_0_H_C_ mT ± 2	µ_0_H_E_ mT ±2	M_S_ A.m^2^.kg^−1^ ±0.5	µ_0_H_C_ mT ± 2	µ_0_H_E_ mT ±2	M_S_ A.m^2^.kg^−1^ ±0.5
*CFO* ceramic	88	0	78.2	—	—	—
*CFO-NO*_*small*_	61	0	49.5	63	0	52.5
*CFO-NO*_*large*_	91	0	46.5	94	0	47.3

## Discussion

In view of the results reported in the Characterization section, it is clear that the composition evolves during the sintering step of the sample preparation. The high energy provided by the SPS pulsed current, temperature and pressure, together with a reductive atmosphere, causes atomic diffusion, involving all the phases introduced as powders into the graphite crucible before sintering. Considering the lattice parameter and the weight ratios obtained by Rietveld analysis and correlating them with the TEM observations and the magnetic measurements, we were able to propose a schematic representation of the atomic movements affecting the composite ceramic samples, and preventing EB at r.t.

**Figure 6:**
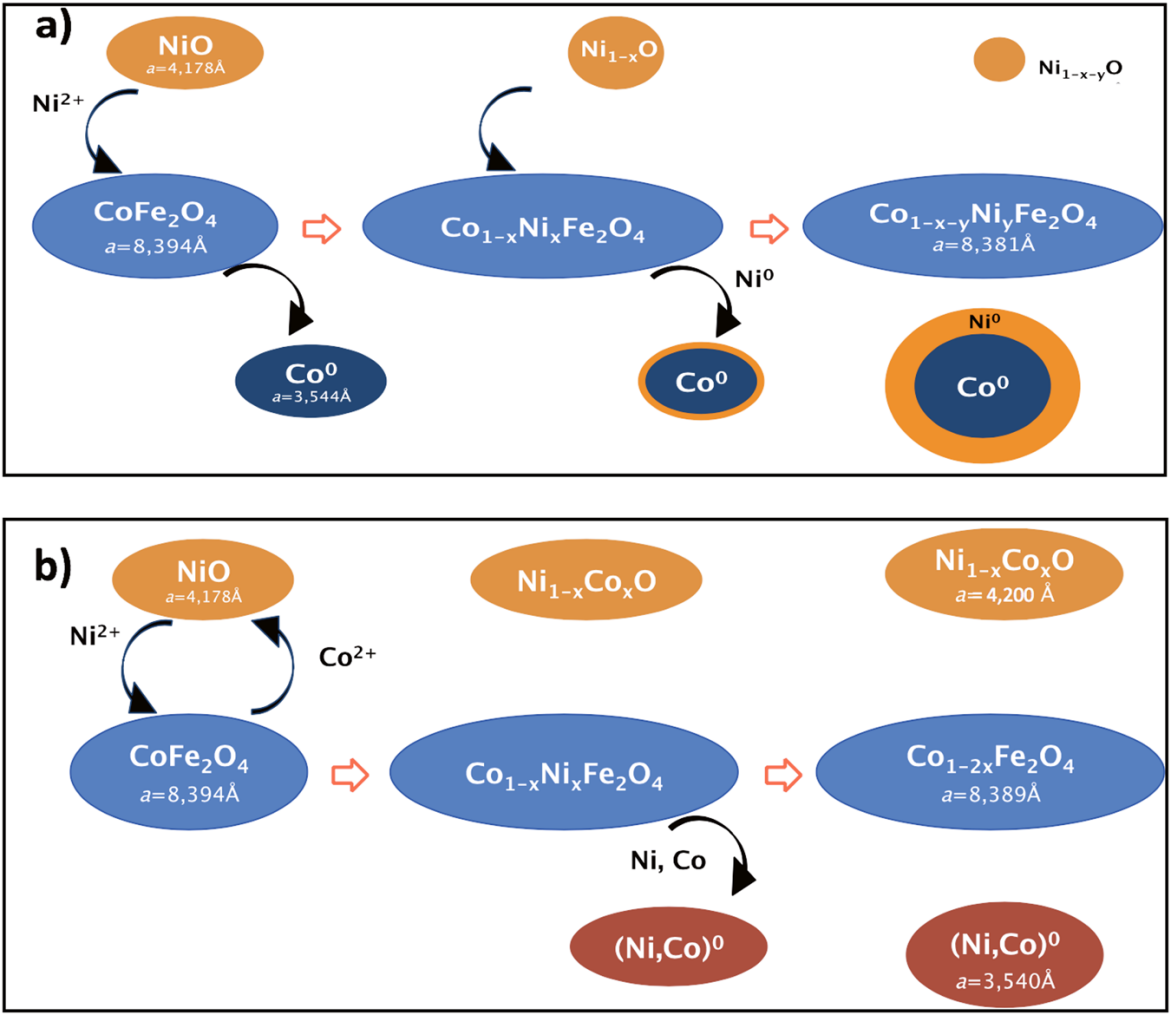
Schematic representation of the atomic diffusion during the preparation of (**a**) *CFO-NO*_small_ and (**b**) *CFO-NO*_large_.

In the case of *CFO-NO*_small_ we propose a general scheme to explain what must happen in the SPS crucible when heating starts (Fig. 6a). We believe that NO particles start to react with the CFO ones. The cations are assumed to diffuse from the NO rock-salt phase into the vacancies of the CFO spinel ones, generated by reduction of a part of the spinel phase Co^2+^ cations into Co° and their expulsion from the F crystallographic lattice. In this way, the spinel phase becomes a mixed Co and Ni ferrite, while the rock-salt phase becomes sub-stoichiometric NiO. Once the Co° cores have been formed, the Ni^2+^ cations freshly inserted into the spinel phase start to be reduced and expulsed, forming a Ni° shell around the Co° cores. The lattice parameter of the spinel phase is then decreased, reaching a value between those of bulk CoFe_2_O_4_ and NiFe_2_O_4_ oxides (see Table 1). This mechanism continues until the almost total disappearance of the rock-salt oxide phase, promoted by its very small size and its very high chemical reactivity. This feature is corroborated by the measured coercive field at r.t. on *CFO-NO*_small_ in comparison with that obtained on pristine *CFO* one (see Table 2). A lower value was obtained for the ceramic in agreement with the partial substitution of Co^2+^ cations into the spinel phase by less anisotropic Ni^2+^ ones.

For *CFO-NO*_large_ different diffusion pathways affect the sample composition (Fig. 6b). At the beginning, a reciprocal diffusion of Ni^2+^ cations from NO into CFO and Co^2+^ from CFO into NO NPs is assumed to take place, during sintering, forming a mixed Co-Ni ferrite as a spinel phase and a mixed Co-Ni monoxide as a rock-salt phase. From this configuration, Co and Ni cations start to be reduced from the spinel phase to metals, forming an alloy of Co and Ni as a metallic phase. At the end of sintering, almost all the Ni^2+^ cations in the spinel phase have been reduced and the chemical composition of the spinel phase is very close to CoFe_2_O_4_. The values found for the lattice parameters are in agreement with this sample composition (see Table 1). This reactional scheme is also supported by the measured coercive field at r.t. on this composite ceramic since a large value close to that of pristine *CFO* was obtained (see Table 2).

Figure 6 summarizes all these SPS induced changes, highlighting the main atomic interdiffusion pathways between the involved phases. In the case of *CFO-NO*_small_ (Fig. 6a) the ratio of the residual AF NiO phase is too small to create an exchange-bias effect after coupling with the F phase. On the other hand, *CFO-NO*_large_ has an AF phase corresponding to a mixed Ni-Co monoxide (Fig. 6b). The presence of Co^2+^ in the rock-salt oxide lattice, even at low concentration, implies a reduction of the Néel temperature of the AF phase^46^, which is probably decreased to a value close to r.t. For this reason the exchange-bias phenomenon cannot be easily detected under our magnetic operating conditions.

**Figure 7:**
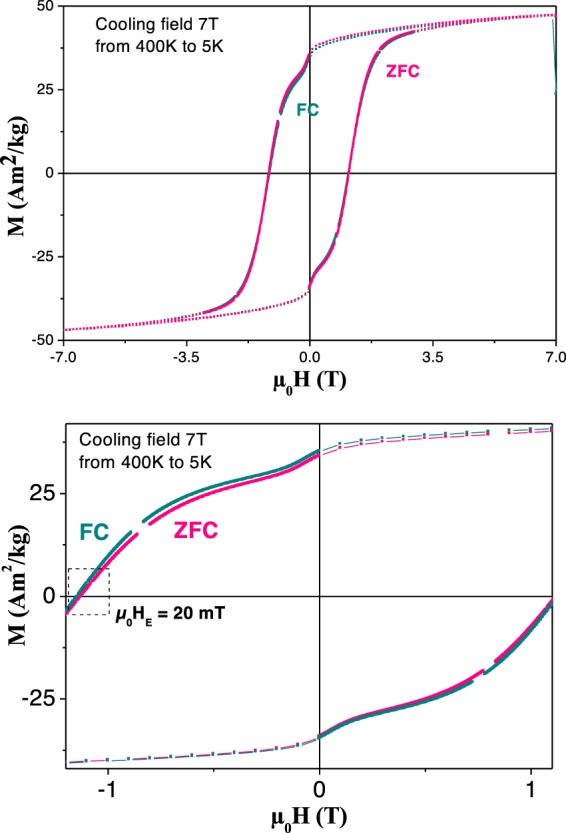
FC and ZFC M(H) hysteresis loops recorded at 5 K, on *CFO-NO*_large_, for an applied magnetic field between +7 and −7 T and a cooling magnetic field of +7 T. The sample was heated to 400 K before being cooled to 5 K in the presence or not of an external magnetic field. A zoom on the plots recorded for a magnetic field varying between −1.1 and +1.1 T is given for clarity.

For these reasons, the hysteresis loops of this sample were specifically measured at 5 K, after a zero-field (ZFC) and a 7 T-field cooling (FC) from a temperature high enough to be considered as above the Néel temperature of its new AF phase. In practice, we cooled the sample from 400 K to 5 K under both FC and ZFC measuring conditions. In both cases, the recorded hysteresis loops are consistent with the superposition of a major hard magnetic contribution (CoFe_2_O_4_ grains) at the origin of the curve broadening along the field axis and a minor soft one (Co_1-x_Ni_x_ grains), characterized by a fast magnetization decrease as H approaches zero. The comparison of the two loops evidences a slight horizontal shift of the FC curve relative to the ZFC one (Fig. 7), leading to an exchange field µ_0_H_E_ of about 20 mT. We suppose that H_E_ mainly results from exchange coupling between the surface spins of CoFe_2_O_4_ and Ni_1-x_Co_x_O grains, the coupling between the surface spins of Co_1-x_Ni_x_ and Ni_1-x_Co_x_O grains, being neglected, as a consequence of the larger size of the metallic grains.

Ultimately, at r.t. the exchange-bias effect was observed in none of the ceramic samples, but for different reasons. In the case of the *NO*_*small*_-derived composite ceramic, the content of AF phase in the final ceramic is almost zero, which is inadequate for EB. In the case of *NO*_*large*_-derived composite ceramic, instead, the weight content of the AF phase does not change significantly during the sintering process but its chemical composition does, resulting in a mixed Ni-Co monoxide with a T_N_ lower than the typical values known for bulk NiO (525 K) and probably close that of bulk CoO (290 K). This fact does not allow the EB effect at r.t., but requires magnetic measurements at lower temperature, which have highlighted the expected EB effect (see Fig. SI-3 in the supporting information section), far below the Neel temperature of the *in situ* formed Ni_1-x_Co_x_O antiferromagnet.

## Conclusions

During the present study, hetero-nano-structured composite ceramics based on oxide components with AF and F magnetic properties were prepared. In particular, the preparation of seed powders by polyol synthesis and their consolidation by the SPS technique allowed to obtain high-density, fine-grained microstructures, with mean grain sizes on the nanometric scale even after the sintering process. Nevertheless, exchange-bias by coupling a ferromagnetic oxide (F) with an antiferromagnetic one (AF) at the nanometric scale was not achieved. Even if the nano-structuration is conserved, the high energy involved in the SPS process, along with the presence of a pulsed DC current and reductive atmosphere, induces atomic diffusion of all the phases. In both samples, a novel metallic phase is found after SPS, but the nature of this phase is different in each sample. In fact, the mechanism of atomic diffusion depends on the nature of the seed powders introduced before sintering. In this specific case, it was observed that the mean size of the NO NPs affects the stability of the NiO phase, both during the sintering process and the magnetic measurements. Moreover, the extent of atomic diffusion seems to be much higher for the smaller NO NPs. However, the NO_large_-derived ceramic is not stable enough for atomic diffusion to be avoided during the consolidation process. Even if a certain amount of the rock-salt phase remains in this ceramic, its composition is different from that of the initial one, thus changing also the characteristic transition temperature T_N_ of the AF phase, that is crucial for observing the EB phenomenon at a given T.

The SPS process turns out to be inappropriate for this kind of application; other types of flash sintering must be investigated, avoiding the application of pulsed DC currents and reductive atmospheres that cause atomic diffusion and composition modification during sintering.

## Materials and Methods

### Chemicals

Fe(CH_3_CO_2_)_2_, Ni(CH_3_CO_2_)_2_.4H_2_O, Co(CH_3_CO_2_)_2_.4H_2_O metal salt precursors and HO(CH_2_)_2_O(CH_2_)_2_OH (DEG, b.p. = 245 °C), HO(CH_2_)_2_O(CH_2_)_2_O(CH_2_)OH (TEG, b.p. = 285 °C) solvents were purchased from ACROS and used without purification.

### CFO NP synthesis

**Figure 8:**
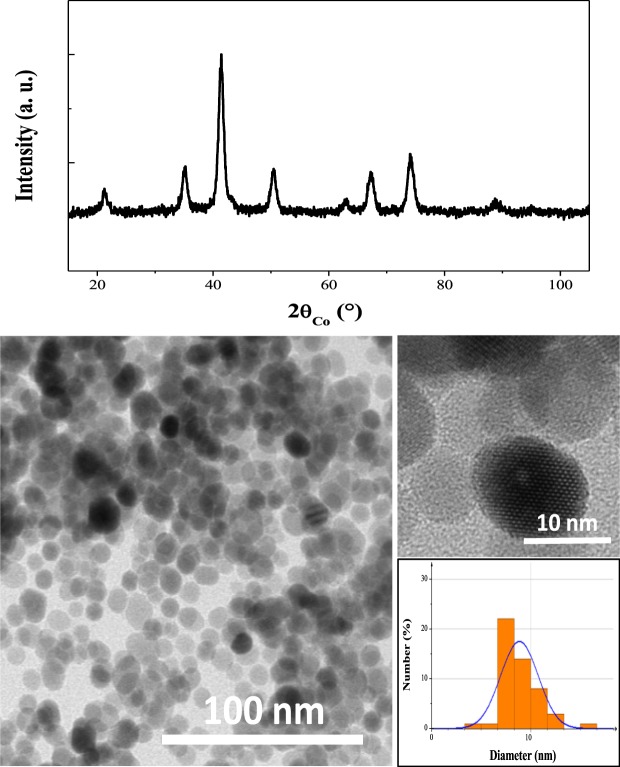
XRD pattern of as-produced 10 nm cobalt ferrite NPs (up) and their TEM micrograph (down). In the inset, a HRTEM image of representative CFO single crystals and a particle size distribution inferred from a statistical analysis of the TEM micrograph using ImageJ software, assuming a spherical shape and fitting the data by a log-normal law.

Well-crystallized, 10 nm cobalt ferrite particles (Fig. 8) were prepared by the polyol method^47^. 12.5 cobalt acetate and 25.0 mmol of iron acetate were dissolved in 125 mL of TEG, and heated under reflux with mechanical stirring for 3 hours. The suspension was allowed to cool with continued stirring; the solids obtained were recovered by centrifugation and washed with ethanol. They were then dried overnight at 50 °C.

### NO NP synthesis

**Figure 9:**
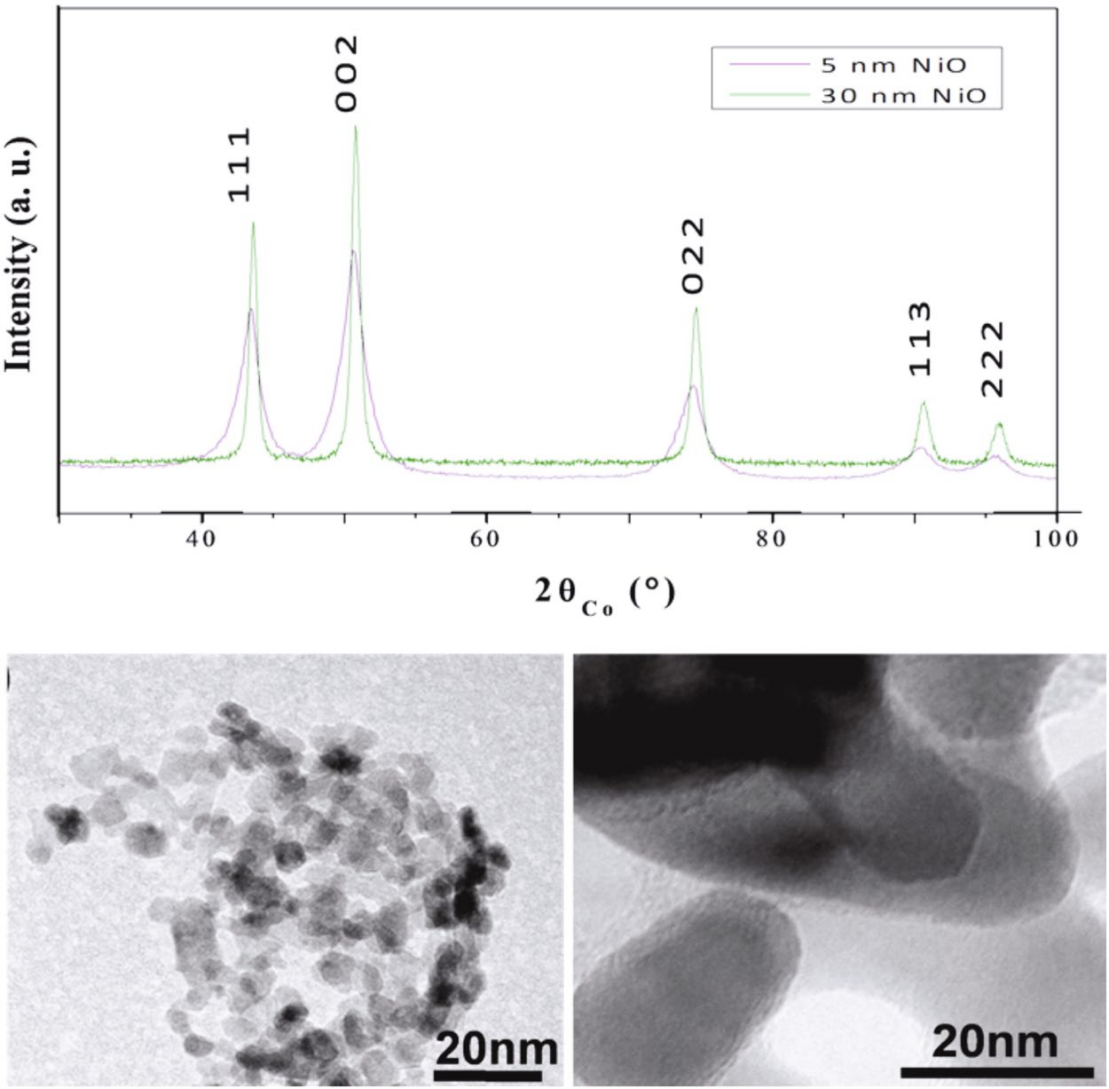
XRD pattern of as-produced 5 and 30 nm nickel oxide particles (up) and their TEM micrographs (down).

5 and 30 nm nickel oxide particles were prepared by combining the polyol process and subsequent air annealing at given temperatures. Typically nickel acetate was dissolved in 250 mL of DEG (0.1 M). A given amount of water was added to the reaction solution to reach a hydrolysis ratio h, defined by the mole ratio of water to nickel, of 12. The mixture was then heated under mechanical stirring up to 180 °C (6 °C. min^−1^) and maintained under reflux and stirring for 3 hours. After rapid cooling to room temperature, a green precipitate formed corresponding to a nickel layered hydroxide salt^48^ which was recovered by centrifugation, washed with ethanol and dried in air overnight. The green solid was then annealed in air at 300 and 600 °C for 2 hours to form the rock-salt NiO phase of different sizes, small and large, respectively (Fig. 9).

### NP sintering

**Figure 10:**
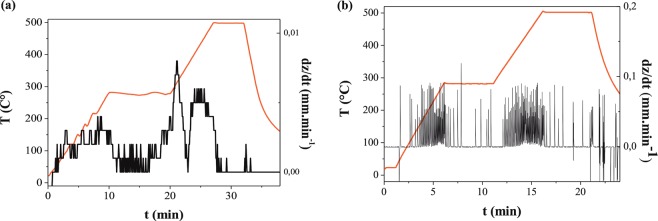
Temperature profile of SPS process (red). The derivative of the piston position dZ/dt is also shown, in black, starting from (**a**) bare CFO NPs or (**b**) a mixture of CFO NPs with 30 nm NO NPs.

Prior to SPS 750 and 250 mg of the as-prepared CFO and NO NPs were treated in an oscillating milling equipment (Retsch MM200) for 5 min at a frequency of 20 Hz, in order to achieve a good mixture. An 8 mm-diameter carbon die with a layer of protective graphite foil (Papyex®) was then filled with the mixture and closed on both sides by carbon pistons. An initial uniaxial pressure of 50 MPa was then applied on the pistons before DC pulse delivery using a DR. SINTER515S SYNTEX SPS machine (Thiais, France). The temperature was first increased to a first plateau of 280 °C, during which the pressure was increased to 100 MPa. The soak time at 280 °C allowed the elimination of any organic residues from the polyol reaction. After this plateau, the temperature was increased to 500 °C and maintained for 5 min (black line in Fig. 10). The derivative of the distance between pistons, dZ/dt, with time, was followed during the heating (black line in Fig. 10) to determine the lowest sintering temperature.

When the distance between pistons shows a significant reduction at constant pressure (volume contraction), then the temperature of sintering is reached. A maximum occurs between 400 and 500 °C, meaning that this range is high enough to reduce significantly the porosity inside the pellets and low enough to limit drastically their average grain growth, leading to the desired highly dense and ultrafine-grained ceramics.

To serve as a reference, nanostructured *CFO* and *NO* ceramics were prepared by SPS, starting from the polyol-prepared NPs and operating under the same sintering conditions.

### Structural and microstructural characterizations

The powder and pellet X-ray diffraction patterns were recorded on a Xpert’Pro (PANALYTICAL) diffractometer equipped with a Co Kα X-ray source (1.7889 Å), in the θ-θ Bragg-Brentano configuration. They were then analysed by Rietveld refinements using MAUD software^49^. Transmission Electron Microscopy was carried out on a JEOL 2100 F microscope operating at 200 kV. The microscope was equipped with a field-emission gun, a high-resolution UHR pole piece and a Gatan US4000 CCD camera. X-ray Energy Dispersive Spectroscopy (XEDS) analyses were also performed by means of a JEOL detector coupled with a scanning TEM device, mounted on the same microscope. Zero-Loss Peak TEM images (TEM-ZLP) were obtained using a GIF2001 Gatan Imaging Filter selecting elastically scattered electrons. Energy-Filtered TEM images were acquired using the three-windows method at the O-K edge, and the Fe-, Co- and Ni-L_2,3_ edges. The powders were prepared by dispersing them in ethanol by sonication and depositing a drop of the resulting suspension on a copper TEM grid. The pellets were prepared by cutting thin foils using the Focused Ion Beam (FIB) technique according to a well-established procedure described elsewhere^50^. The foils were then deposited on a classical TEM grid for the observations.

### Magnetic characterization

The ZFC and FC thermal variations of the magnetization of the powders and ceramics produced were plotted using a Lake Shore VSM instrument with a 1.4 T magnetizing field, equipped with a head that can heat the sample under Ar flow to 950 K (*Laboratoire SATIE- ENS Cachan*). The r.t. ZFC and FC variations of their magnetization as a function of the applied field were also plotted. Each sample was analyzed by following a precise procedure, with the aim of inducing exchange coupling between the spins at the AF-F interface. To do this, a relatively strong external magnetic field (1.4 T) was applied to the samples at 600 K, a temperature between the T_C_ value of CoFe_2_O_4_ and the T_N_ value of NiO, and then maintained during the cooling step. The same set of experiments was carried out on pristine *CFO*, *NO*_small_ and *NO*_large_ ceramics to serve as references. Before the samples were magnetized for the first time, a hysteresis M(H) loop between −1.4 T and 1.4 T was measured at 300 K on the pristine and composite ceramics. The sample magnetization was then recorded at increasing temperatures from 300 up to 600 K under 1.4 T applied magnetic field. In the same way, the magnetization was recorded during the cooling phase, always with a 1.4 T applied field, and a hysteresis loop at 300 K was finally measured at the end of the heating-cooling cycle. To be as exhaustive as possible, the hysteresis loops of the composite ceramics were also measured at 5 K using a Quantum Design PPMS magnetometer in both the FC and ZFC modes, starting from 400 K for a cooling magnetic field of 7 T.

## Supplementary information

Supplementary Information
