# Supplementary material for: On the limits of Reactive-Spark-Plasma Sintering to prepare magnetically enhanced nanostructured ceramics: the case of the CoFe2O4-NiO system
Source: Sci Rep. 2019 Oct 1;9:14119. doi: 10.1038/s41598-019-50657-4 (PMC6773756; doi:10.1038/s41598-019-50657-4)
Supplement: Supplementary file 1 — Supplementary Information [file 41598_2019_50657_MOESM1_ESM.docx]

**SUPPLEMENTARY INFORMATION**

On the limits of Reactive-Spark-Plasma Sintering to prepare magnetically enhanced nanostructured ceramics: the case of the CoFe_2_O_4_-NiO system

**Giulia Franceschin^1,2*^, Thomas Gaudisson^1,2^, Nicolas Menguy^3^, Raul Valenzuela^4^, Frederic Mazaleyrat^2^, Souad Ammar ^1^**^*^

^1^ ITODYS, Université Paris Diderot, Sorbonne Paris Université, CNRS UMR-7086, 75013 Paris, France.

^2^ SATIE, ENS Cachan, Paris Saclay, CNRS UMR-8029, 94235 Cachan, France.

^3^ Sorbonne Université, UMR CNRS 7590, MNHN, IRD, Institut de Minéralogie, de Physique des Matériaux et de Cosmochimie, IMPMC, 75005 Paris, France.

^4^ IMM, Universidad Nacional Autónoma de Mexico, 04510 Mexico City, Mexico.

* [giulia.franceschin@univ-paris-diderot.fr](mailto:giulia.franceschin@univ-paris-diderot.fr), ammarmer@univ-paris-diderot.fr

Figure SI-1. Experimental (scatter) and calculated (red solid line) XRD patterns of A. *CFO-NO* ceramics prepared starting from small and large NO particles, and pristine *CFO* ceramic and B. *NO* ceramics prepared starting from small and large NO particles. The difference between the experimental and calculated diffractograms (blue line) illustrates the fit quality. The Bragg reliability factor R_B_ ranges between 1 and 2 for all the performed refinements.

Figure SI-2. Histograms representing the grain size distribution of all composite ceramics and their *NO* reference ones as inferred from SEM analysis. The grains are considered as spheres and the diameter of about 100 grains was taken into account for each sample. The average diameter and the standard deviation are given on the histograms.

Figure SI-3. FC Hysteresis cycles recorded for the sample *CIO-NO_large_* ceramic at incresing temperature, from 50 K to 300 K. The applied magnetic field ranges between +7 and -7 T and the cooling magnetic field is +7 T. The sample was heated up to 400 K before being cooled down to 50 K in presence of 7 T magnetic field. A zoom on the recorded plots around 0 axes and a µ_0_H_E_ vs T trend are given, showing a decrease of the exchange field when the temperature increases, as commonly observed in E-biased structures.
